# Tetra­ethyl­ammonium (acetyl­acetonato)bromidotricarbonyl­rhenate(I)

**DOI:** 10.1107/S1600536810050105

**Published:** 2010-12-04

**Authors:** Alice Brink, Hendrik G. Visser, Andreas Roodt

**Affiliations:** a Department of Chemistry, University of the Free State, PO Box 339, Bloemfontein 9300, South Africa

## Abstract

In the title compound, (C_8_H_20_N)[ReBr(C_5_H_7_O_2_)(CO)_3_], the Re^I^ atom in the rhenate anion is surrounded by three carbonyl ligands orientated in a facial arrangement, a bromide ligand and an acetyl­acetonate ligand, leading to a distorted octa­hedral ReC_3_BrO_2_ coordination with a O—Re—O bite angle of 85.66 (7)°. An array of C—H⋯O and C—H⋯Br hydrogen-bonding inter­actions between the cations and the surrounding rhenate anions stabilize the crystal structure.

## Related literature

For the synthesis of the Re(I)–tricarbonyl synthon, see: Alberto *et al.* (1996 ▶). For related rhenium–tricarbonyl complexes, see: Mundwiler *et al.* (2004 ▶); Wang *et al.* (2003 ▶); Saw *et al.* (2006 ▶). For studies of related rhenium(V) compounds, see: Roodt *et al.* (1992 ▶); Purcell *et al.* (1989 ▶). For acetyl­acetonato complexes and related structures, see: Brink *et al.* (2007*a* ▶,*b* ▶; 2010 ▶); Steyl & Hill (2009 ▶); Herbst *et al.* (2010 ▶). For a rhenium complex with pyridine and acetyl­acetonato ligands, see: Benny *et al.* (2008 ▶). For related structures, see: Schutte *et al.* (2009 ▶, 2010 ▶).
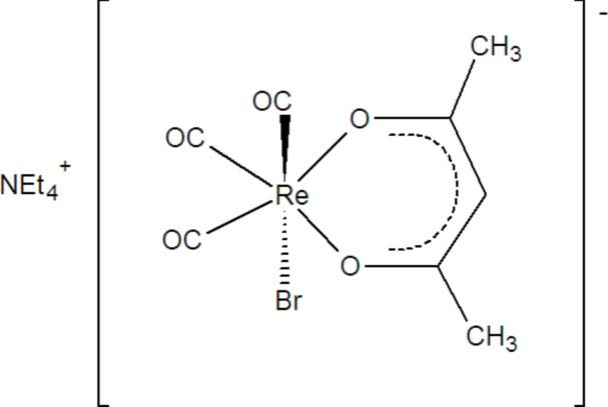


## Experimental

### 

#### Crystal data

(C_8_H_20_N)[ReBr(C_5_H_7_O_2_)(CO)_3_]
*M* *_r_* = 579.5
Orthorhombic, 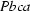

*a* = 13.0931 (1) Å
*b* = 14.5865 (1) Å
*c* = 20.8724 (2) Å
*V* = 3986.26 (6) Å^3^
*Z* = 8
Mo *K*α radiation
μ = 8.12 mm^−1^
*T* = 100 K
0.26 × 0.13 × 0.08 mm

#### Data collection

Oxford Diffraction Xcalibur3 CCD diffractometer
Absorption correction: multi-scan (*CrysAlis RED*; Oxford Diffraction, 2006 ▶) *T*_min_ = 0.227, *T*_max_ = 0.563
30330 measured reflections
4819 independent reflections
3641 reflections with *I* > 2σ(*I*)
*R*_int_ = 0.031

#### Refinement

*R*[*F* ^2^ > 2σ(*F* ^2^)] = 0.019
*wR*(*F* ^2^) = 0.047
*S* = 1.02
4819 reflections
223 parameters
H-atom parameters constrained
Δρ_max_ = 1.35 e Å^−3^
Δρ_min_ = −0.71 e Å^−3^

### 

Data collection: *CrysAlis CCD* (Oxford Diffraction, 2006 ▶); cell refinement: *CrysAlis RED* (Oxford Diffraction, 2006 ▶); data reduction: *CrysAlis RED*; program(s) used to solve structure: *SHELXS97* (Sheldrick, 2008 ▶); program(s) used to refine structure: *SHELXL97* (Sheldrick, 2008 ▶); molecular graphics: *DIAMOND* (Brandenburg & Putz, 2004 ▶); software used to prepare material for publication: *WinGX* (Farrugia, 1999 ▶).

## Supplementary Material

Crystal structure: contains datablocks global, I. DOI: 10.1107/S1600536810050105/wm2432sup1.cif

Structure factors: contains datablocks I. DOI: 10.1107/S1600536810050105/wm2432Isup2.hkl

Additional supplementary materials:  crystallographic information; 3D view; checkCIF report

## Figures and Tables

**Table 1:** Hydrogen-bond geometry (Å, °)

*D*—H⋯*A*	*D*—H	H⋯*A*	*D*⋯*A*	*D*—H⋯*A*
C31—H31*A*⋯O01^i^	0.99	2.5	3.378 (4)	147
C31—H31*B*⋯O01	0.99	2.58	3.543 (4)	165
C35—H35*B*⋯O03^ii^	0.99	2.54	3.221 (3)	126
C36—H36*B*⋯O03^ii^	0.98	2.57	3.155 (4)	118
C37—H37*A*⋯Br1^iii^	0.99	2.91	3.859 (3)	161

Symmetry codes: (i) 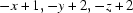; (ii) 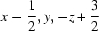; (iii) 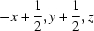.

**Fig. 1.**
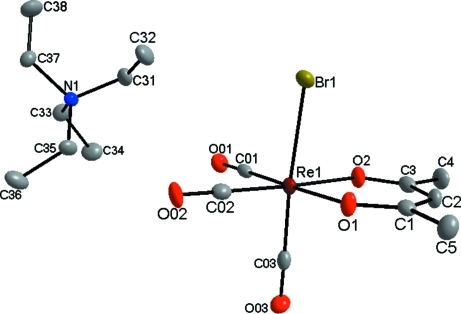
Representation of the molecular structure of the title compound, showing the numbering scheme and displacement ellipsoids drawn at 50% probability level. Hydrogen atoms are omitted for clarity.

**Fig. 2.**
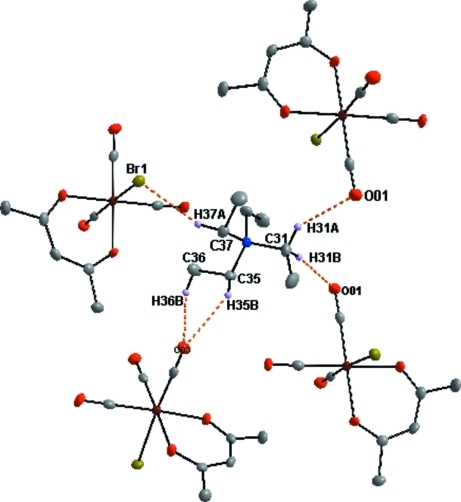
Representation of the hydrogen-bonding interactions.

## supplementary crystallographic information

### Comment

The title compound forms part of an ongoing investigation aimed at determining the crystallographic and kinetic effects experienced by Re(I) and Re(V) complexes (Roodt *et al.*, 1992, Purcell *et al.*, 1989), in particular the manner which various *O, O'*-bidentate ligands have on rhenium tricarbonyl complexes as well as other transition group metals such as rhodium (Brink *et al.*, 2010), silver (Steyl & Hill, 2009) and niobium (Herbst *et al.*, 2010). Various rhenium tricarbonyl bidentate ligands have been synthesized (Mundwiler *et al.*, 2004, Wang *et al.*, 2003, Saw *et al.*, 2006), however few *O, O'*-bidentate ligands are reported in literature (Schutte *et al.*, 2010).

The octahedral geometry around the Re(I) metal atom in the rhenate anion shows little distortion (Fig. 1) with an O1—Re—O2 bite angle of 85.66 (7)°, which correlates well with a pyridine-coordinated rhenium acetylacetonato complex (85.07 (8)°; Benny *et al.*, 2008) and is similar to rhodium acetylacetonato complexes (88.69 (8)° and 88.20 (6)°; Brink *et al.*, 2007*a*,*b*). The Re—O_acac_ bond lengths (acac is acetylacetonate) of the title compound (2.1248 (18) Å and 2.1265 (19) Å) are slightly longer than that found in the pyridine analogue (2.1189 (19) Å and 2.1226 (19) Å; Benny *et al.*, 2008). The Re—Br bond lengths of 2.6448 (3) Å compares well with related structures (Schutte *et al.*, 2009, 2010). Intermolecular C—H···O and C—H···Br hydrogen-bonding interactions are observed between rhenate anions and neighboring cations (Table 1 and Fig. 2)

### Experimental

[NEt_4_]_2_[Re(CO)_3_Br_3_] (0.13 mmol) (synthesized according to Alberto *et al.* (1996)) was dissolved in 6 ml methanol. Acetylacetone (0.14 mmol), dissolved in 6 ml methanol was slowly added. The reaction mixture was heated to 329 K for 24 h. Crystals of the title complex were obtained by the slow evaporation of the solvent. Colourless crystals were stable in air for several months.

### Refinement

The H atoms were placed in geometrically idealized positions and constrained to ride on their parent atoms with *U*_iso_(H) = 1.2*U*_eq_(C) and 1.5*U*_eq_(C) for the methine, methylene and methyl carbon atoms, respectively. The methyl groups were generated to fit the difference electron density and the groups were then refined as rigid rotors. The highest peak and deepest hole in the final difference map are located 1.12Å and 0.61Å from Br1 and H33*a*, respectively.

### Crystal data

**Table:**

(C_8_H_20_N)[ReBr(C_5_H_7_O_2_)(CO)_3_]	*F*(000) = 2240
*M**_r_* = 579.5	*D*_x_ = 1.931 Mg m^−^^3^
Orthorhombic, *P**b**c**a*	Mo *K*α radiation, λ = 0.71073 Å
Hall symbol: -P 2ac 2ab	Cell parameters from 16272 reflections
*a* = 13.0931 (1) Å	θ = 2.3–33.0°
*b* = 14.5865 (1) Å	µ = 8.12 mm^−^^1^
*c* = 20.8724 (2) Å	*T* = 100 K
*V* = 3986.26 (6) Å^3^	Parallelepiped, colourless
*Z* = 8	0.26 × 0.13 × 0.08 mm

### Data collection

**Table:**

Oxford Diffraction Xcalibur3 CCD diffractometer	4819 independent reflections
Radiation source: Enhance (Mo) X-ray Source	3641 reflections with *I* > 2σ(*I*)
graphite	*R*_int_ = 0.031
Detector resolution: 16.1829 pixels mm^-1^	θ_max_ = 28°, θ_min_ = 2.3°
ω–scans	*h* = −17→16
Absorption correction: multi-scan (*CrysAlis RED*; Oxford Diffraction, 2006)	*k* = −19→15
*T*_min_ = 0.227, *T*_max_ = 0.563	*l* = −27→27
30330 measured reflections	

### Refinement

**Table:**

Refinement on *F*^2^	0 restraints
Least-squares matrix: full	H-atom parameters constrained
*R*[*F*^2^ > 2σ(*F*^2^)] = 0.019	*w* = 1/[σ^2^(*F*_o_^2^) + (0.0251*P*)^2^] where *P* = (*F*_o_^2^ + 2*F*_c_^2^)/3
*wR*(*F*^2^) = 0.047	(Δ/σ)_max_ = 0.002
*S* = 1.02	Δρ_max_ = 1.35 e Å^−^^3^
4819 reflections	Δρ_min_ = −0.71 e Å^−^^3^
223 parameters	

### Special details

**Table:**

Experimental. The intensity data was collected on a Oxford Diffraction Xcalibur 3 area detector diffractometer using an exposure time of 10 s/frame. A total of 552 frames were collected with a frame width of 0.75° covering up to θ = 28.00° with 100.0% completeness accomplished.
Geometry. All e.s.d.'s (except the e.s.d. in the dihedral angle between two l.s. planes) are estimated using the full covariance matrix. The cell e.s.d.'s are taken into account individually in the estimation of e.s.d.'s in distances, angles and torsion angles; correlations between e.s.d.'s in cell parameters are only used when they are defined by crystal symmetry. An approximate (isotropic) treatment of cell e.s.d.'s is used for estimating e.s.d.'s involving l.s. planes.

### Fractional atomic coordinates and isotropic or equivalent isotropic displacement parameters (Å^2^)

**Table:**

	*x*	*y*	*z*	*U*_iso_*/*U*_eq_	
C1	0.6479 (2)	0.59694 (18)	0.79693 (14)	0.0171 (6)	
C02	0.4190 (2)	0.79609 (18)	0.82159 (14)	0.0178 (6)	
C01	0.5566 (2)	0.91066 (19)	0.86140 (14)	0.0170 (6)	
C2	0.7475 (2)	0.62159 (19)	0.81179 (14)	0.0198 (6)	
H2	0.7987	0.5764	0.8052	0.024*	
C3	0.7799 (2)	0.70650 (19)	0.83555 (14)	0.0186 (6)	
C03	0.5822 (2)	0.82991 (18)	0.74731 (16)	0.0174 (6)	
C4	0.8919 (2)	0.7204 (2)	0.84831 (16)	0.0253 (7)	
H4A	0.9119	0.7822	0.8349	0.038*	
H4B	0.9315	0.675	0.8242	0.038*	
H4C	0.9054	0.713	0.8942	0.038*	
C5	0.6255 (2)	0.50244 (18)	0.77154 (16)	0.0231 (7)	
H5A	0.5809	0.4699	0.8016	0.035*	
H5B	0.6896	0.4685	0.7666	0.035*	
H5C	0.5915	0.5074	0.7299	0.035*	
C31	0.3312 (2)	0.9931 (2)	0.97904 (15)	0.0247 (7)	
H31A	0.3463	1.0204	1.0214	0.03*	
H31B	0.391	1.0043	0.9511	0.03*	
C32	0.3183 (3)	0.88969 (19)	0.98731 (17)	0.0318 (8)	
H32A	0.2625	0.8776	1.0175	0.048*	
H32B	0.3818	0.8633	1.004	0.048*	
H32C	0.3022	0.8618	0.9458	0.048*	
C33	0.2623 (2)	1.14451 (18)	0.94940 (15)	0.0214 (6)	
H33A	0.1989	1.1775	0.9377	0.026*	
H33B	0.2816	1.1642	0.9931	0.026*	
C34	0.3465 (2)	1.1732 (2)	0.90338 (17)	0.0304 (8)	
H34A	0.4098	1.141	0.9144	0.046*	
H34B	0.3574	1.2395	0.9066	0.046*	
H34C	0.3265	1.1576	0.8595	0.046*	
C35	0.2202 (2)	1.00610 (19)	0.88378 (14)	0.0191 (6)	
H35A	0.2853	1.0071	0.8597	0.023*	
H35B	0.1979	0.9414	0.887	0.023*	
C36	0.1410 (3)	1.0589 (2)	0.84595 (15)	0.0273 (7)	
H36A	0.0767	1.0606	0.87	0.041*	
H36B	0.1297	1.0286	0.8046	0.041*	
H36C	0.1652	1.1216	0.8387	0.041*	
C37	0.1443 (2)	1.02603 (19)	0.99096 (14)	0.0190 (6)	
H37A	0.0881	1.0637	0.9733	0.023*	
H37B	0.1242	0.9609	0.9868	0.023*	
C38	0.1560 (3)	1.0482 (2)	1.06086 (15)	0.0302 (8)	
H38A	0.2073	1.0075	1.0799	0.045*	
H38B	0.0904	1.0394	1.0826	0.045*	
H38C	0.1779	1.1121	1.0656	0.045*	
N1	0.23955 (16)	1.04213 (14)	0.95068 (11)	0.0148 (5)	
O1	0.56968 (13)	0.64852 (12)	0.80201 (11)	0.0186 (4)	
O02	0.33086 (15)	0.80287 (13)	0.81550 (11)	0.0234 (5)	
O2	0.72326 (14)	0.77461 (13)	0.84811 (10)	0.0201 (5)	
O01	0.54896 (15)	0.98593 (13)	0.87843 (11)	0.0240 (5)	
O03	0.59154 (15)	0.85547 (14)	0.69515 (11)	0.0240 (5)	
Re1	0.563458 (8)	0.787480 (7)	0.832299 (6)	0.01437 (4)	
Br1	0.54691 (2)	0.718131 (18)	0.948917 (14)	0.01961 (7)	

### Atomic displacement parameters (Å^2^)

**Table:**

	*U*^11^	*U*^22^	*U*^33^	*U*^12^	*U*^13^	*U*^23^
C1	0.0202 (15)	0.0158 (14)	0.0154 (16)	−0.0001 (11)	0.0013 (12)	0.0039 (11)
C02	0.0237 (16)	0.0141 (14)	0.0155 (16)	−0.0029 (11)	0.0002 (12)	0.0010 (11)
C01	0.0139 (14)	0.0209 (15)	0.0162 (15)	−0.0023 (12)	−0.0018 (12)	0.0059 (12)
C2	0.0169 (14)	0.0190 (14)	0.0235 (17)	0.0062 (12)	0.0028 (13)	0.0006 (12)
C3	0.0140 (13)	0.0214 (15)	0.0206 (16)	−0.0013 (11)	0.0009 (12)	0.0064 (13)
C03	0.0118 (14)	0.0137 (13)	0.0267 (18)	0.0000 (11)	−0.0001 (12)	−0.0026 (13)
C4	0.0136 (14)	0.0275 (17)	0.035 (2)	0.0016 (13)	−0.0001 (13)	0.0016 (14)
C5	0.0241 (16)	0.0174 (15)	0.0277 (18)	0.0004 (12)	0.0030 (13)	−0.0031 (13)
C31	0.0179 (15)	0.0329 (17)	0.0233 (18)	0.0056 (13)	−0.0029 (13)	0.0012 (13)
C32	0.0355 (19)	0.0301 (17)	0.0298 (19)	0.0142 (15)	−0.0017 (16)	0.0071 (14)
C33	0.0214 (15)	0.0176 (14)	0.0253 (18)	−0.0068 (12)	0.0006 (13)	−0.0039 (13)
C34	0.0313 (19)	0.0287 (17)	0.031 (2)	−0.0122 (14)	0.0023 (15)	0.0035 (14)
C35	0.0246 (15)	0.0176 (14)	0.0152 (16)	0.0018 (12)	−0.0013 (13)	−0.0023 (12)
C36	0.0371 (19)	0.0215 (16)	0.0233 (19)	−0.0005 (14)	−0.0082 (14)	0.0029 (13)
C37	0.0164 (14)	0.0196 (14)	0.0210 (16)	−0.0005 (11)	0.0043 (12)	0.0015 (12)
C38	0.0336 (19)	0.0355 (19)	0.0213 (19)	0.0006 (15)	0.0078 (14)	0.0000 (14)
N1	0.0144 (12)	0.0151 (11)	0.0148 (13)	−0.0007 (9)	−0.0016 (10)	0.0001 (10)
O1	0.0115 (10)	0.0127 (9)	0.0317 (12)	−0.0014 (8)	−0.0013 (9)	−0.0057 (9)
O02	0.0139 (11)	0.0205 (11)	0.0360 (14)	0.0040 (8)	−0.0083 (9)	−0.0017 (9)
O2	0.0102 (9)	0.0186 (10)	0.0316 (13)	−0.0001 (8)	−0.0021 (8)	−0.0019 (9)
O01	0.0265 (12)	0.0162 (10)	0.0294 (13)	−0.0013 (9)	−0.0042 (10)	−0.0018 (9)
O03	0.0256 (11)	0.0230 (11)	0.0236 (12)	−0.0028 (9)	0.0054 (10)	−0.0003 (10)
Re1	0.01154 (6)	0.01270 (6)	0.01887 (7)	−0.00035 (4)	−0.00109 (4)	−0.00012 (5)
Br1	0.02132 (15)	0.01855 (13)	0.01895 (16)	−0.00067 (12)	−0.00405 (11)	0.00209 (12)

### Geometric parameters (Å, °)

**Table:**

C1—O1	1.275 (3)	C32—H32C	0.98
C1—C2	1.388 (4)	C33—C34	1.521 (4)
C1—C5	1.505 (4)	C33—N1	1.523 (3)
C02—O02	1.165 (3)	C33—H33A	0.99
C02—Re1	1.909 (3)	C33—H33B	0.99
C01—O01	1.158 (3)	C34—H34A	0.98
C01—O01	1.158 (3)	C34—H34B	0.98
C01—Re1	1.899 (3)	C34—H34C	0.98
C2—C3	1.400 (4)	C35—C36	1.513 (4)
C2—H2	0.95	C35—N1	1.513 (4)
C3—O2	1.267 (3)	C35—H35A	0.99
C3—C4	1.504 (4)	C35—H35B	0.99
C03—O03	1.157 (4)	C36—H36A	0.98
C03—Re1	1.895 (3)	C36—H36B	0.98
C4—H4A	0.98	C36—H36C	0.98
C4—H4B	0.98	C37—C38	1.502 (4)
C4—H4C	0.98	C37—N1	1.522 (3)
C5—H5A	0.98	C37—H37A	0.99
C5—H5B	0.98	C37—H37B	0.99
C5—H5C	0.98	C38—H38A	0.98
C31—N1	1.517 (3)	C38—H38B	0.98
C31—C32	1.528 (4)	C38—H38C	0.98
C31—H31A	0.99	O1—Re1	2.1248 (18)
C31—H31B	0.99	O2—Re1	2.1265 (19)
C32—H32A	0.98	Re1—Br1	2.6448 (3)
C32—H32B	0.98		
			
O1—C1—C2	125.7 (3)	H34B—C34—H34C	109.5
O1—C1—C5	114.4 (2)	C36—C35—N1	114.8 (2)
C2—C1—C5	119.9 (3)	C36—C35—H35A	108.6
O02—C02—Re1	178.8 (2)	N1—C35—H35A	108.6
O01—C01—Re1	177.7 (2)	C36—C35—H35B	108.6
O01—C01—Re1	177.7 (2)	N1—C35—H35B	108.6
C1—C2—C3	126.4 (3)	H35A—C35—H35B	107.5
C1—C2—H2	116.8	C35—C36—H36A	109.5
C3—C2—H2	116.8	C35—C36—H36B	109.5
O2—C3—C2	126.1 (3)	H36A—C36—H36B	109.5
O2—C3—C4	115.4 (2)	C35—C36—H36C	109.5
C2—C3—C4	118.5 (3)	H36A—C36—H36C	109.5
O03—C03—Re1	178.6 (3)	H36B—C36—H36C	109.5
C3—C4—H4A	109.5	C38—C37—N1	114.8 (2)
C3—C4—H4B	109.5	C38—C37—H37A	108.6
H4A—C4—H4B	109.5	N1—C37—H37A	108.6
C3—C4—H4C	109.5	C38—C37—H37B	108.6
H4A—C4—H4C	109.5	N1—C37—H37B	108.6
H4B—C4—H4C	109.5	H37A—C37—H37B	107.5
C1—C5—H5A	109.5	C37—C38—H38A	109.5
C1—C5—H5B	109.5	C37—C38—H38B	109.5
H5A—C5—H5B	109.5	H38A—C38—H38B	109.5
C1—C5—H5C	109.5	C37—C38—H38C	109.5
H5A—C5—H5C	109.5	H38A—C38—H38C	109.5
H5B—C5—H5C	109.5	H38B—C38—H38C	109.5
N1—C31—C32	115.0 (2)	C35—N1—C31	109.2 (2)
N1—C31—H31A	108.5	C35—N1—C37	108.6 (2)
C32—C31—H31A	108.5	C31—N1—C37	111.1 (2)
N1—C31—H31B	108.5	C35—N1—C33	110.9 (2)
C32—C31—H31B	108.5	C31—N1—C33	108.3 (2)
H31A—C31—H31B	107.5	C37—N1—C33	108.7 (2)
C31—C32—H32A	109.5	C1—O1—Re1	128.19 (17)
C31—C32—H32B	109.5	C3—O2—Re1	127.81 (18)
H32A—C32—H32B	109.5	C03—Re1—C01	89.80 (12)
C31—C32—H32C	109.5	C03—Re1—C02	89.85 (12)
H32A—C32—H32C	109.5	C01—Re1—C02	85.88 (11)
H32B—C32—H32C	109.5	C03—Re1—O1	91.61 (10)
C34—C33—N1	115.0 (2)	C01—Re1—O1	178.54 (10)
C34—C33—H33A	108.5	C02—Re1—O1	93.78 (9)
N1—C33—H33A	108.5	C03—Re1—O2	92.67 (10)
C34—C33—H33B	108.5	C01—Re1—O2	94.63 (9)
N1—C33—H33B	108.5	C02—Re1—O2	177.43 (10)
H33A—C33—H33B	107.5	O1—Re1—O2	85.66 (7)
C33—C34—H34A	109.5	C03—Re1—Br1	175.69 (8)
C33—C34—H34B	109.5	C01—Re1—Br1	93.65 (9)
H34A—C34—H34B	109.5	C02—Re1—Br1	92.97 (9)
C33—C34—H34C	109.5	O1—Re1—Br1	84.96 (6)
H34A—C34—H34C	109.5	O2—Re1—Br1	84.49 (6)
			
O1—C1—C2—C3	0.8 (5)	C34—C33—N1—C31	67.4 (3)
C5—C1—C2—C3	179.9 (3)	C34—C33—N1—C37	−171.7 (2)
C1—C2—C3—O2	0.9 (5)	C2—C1—O1—Re1	1.3 (4)
C1—C2—C3—C4	−179.5 (3)	C5—C1—O1—Re1	−177.82 (19)
C36—C35—N1—C31	−171.2 (2)	C2—C3—O2—Re1	−4.3 (4)
C36—C35—N1—C37	67.6 (3)	C4—C3—O2—Re1	176.09 (19)
C36—C35—N1—C33	−51.8 (3)	C1—O1—Re1—C03	89.5 (3)
C32—C31—N1—C35	−62.4 (3)	C1—O1—Re1—C02	179.5 (2)
C32—C31—N1—C37	57.3 (3)	C1—O1—Re1—O2	−3.0 (2)
C32—C31—N1—C33	176.6 (2)	C1—O1—Re1—Br1	−87.9 (2)
C38—C37—N1—C35	173.2 (2)	C3—O2—Re1—C03	−86.9 (2)
C38—C37—N1—C31	53.1 (3)	C3—O2—Re1—C01	−177.0 (2)
C38—C37—N1—C33	−66.1 (3)	C3—O2—Re1—O1	4.5 (2)
C34—C33—N1—C35	−52.4 (3)	C3—O2—Re1—Br1	89.8 (2)

### Hydrogen-bond geometry (Å, °)

**Table:**

*D*—H···*A*	*D*—H	H···*A*	*D*···*A*	*D*—H···*A*
C31—H31A···O01^i^	0.99	2.5	3.378 (4)	147.
C31—H31B···O01	0.99	2.58	3.543 (4)	165.
C35—H35B···O03^ii^	0.99	2.54	3.221 (3)	126.
C36—H36B···O03^ii^	0.98	2.57	3.155 (4)	118
C37—H37A···Br1^iii^	0.99	2.91	3.859 (3)	161.

Symmetry codes: (i) −*x*+1, −*y*+2, −*z*+2;  (ii) *x*−1/2, *y*, −*z*+3/2; (iii) −*x*+1/2, *y*+1/2, *z*.
